# Electronic Chip‐Mimetic Medical Implants for Controllable Sonothermal Treatments

**DOI:** 10.1002/advs.77206

**Published:** 2026-08-15

**Authors:** Zhengdong Zhang, Pan Liu, Jie Lei, Shuchang Peng, Qiu Chen, Wei Guan, Yizhou Zhu, Gang Liu, Xiaobo Feng, Cao Yang, Lei Tan

**Affiliations:** ^1^ Department of Orthopaedics, Union Hospital, Tongji Medical College Huazhong University of Science and Technology Wuhan People's Republic of China; ^2^ School of Clinical Medicine, Chengdu Medical College, Department of Orthopedics, Development and Regeneration Key Laboratory of Sichuan Province The First Affiliated Hospital of Chengdu Medical College Chengdu People's Republic of China; ^3^ Clinical Medical College & Affiliated Hospital of Chengdu University Chengdu People's Republic of China; ^4^ Department of Spine Surgery, Guangdong Provincial People's Hospital (Guangdong Academy of Medical Sciences) Southern Medical University Guangzhou People's Republic of China; ^5^ Hospital of Chengdu University of Traditional Chinese Medicine Chengdu People's Republic of China; ^6^ Department of Orthopaedics & Traumatology, Li Ka Shing Faculty of Medicine The University of Hong Kong Pokfulam Hong Kong People's Republic of China

**Keywords:** electronic chip‐mimetic, infection/osseointegration, medical implants, sonothermal treatments, thrombolysis

## Abstract

The sterility and controllable biological functionalities of implantable medical devices determine their lifespan, safety, and therapeutic efficacy. Inspired by the thermal effect of working electronic chips, which consist of various heterostructures, we have developed an electronic chip‐mimetic sonothermal platform through constructing metal‐semiconductor heterogeneous interface. Through magnetron sputtering on the surface of the sandblasted/acid‐etched pretreated titanium (pTi), we find that semiconductor coatings (TiO_2_, Si, ZnO, and Te) endow pTi with different in situ sonothermal effects (ΔT > 18°C, 15 min) under ultrasound (US) irradiation, whereas conductor coatings do not. The sonothermal mechanism of pTi‐semiconductor is associated with US‐activated electron and phonon transport within the heterogeneous interface of implants, which is determined by the electrical and phonon characteristics of pTi‐semiconductor, including their matching degree, thermal conductivity, defected structure, and type of semiconductor. Clinical titanium screws were introduced with defected structure in oxygen layer (TiO_2‐x_) and bone‐derived whitlockite, which shows great sonothermal/sonodynamic effects for efficient elimination of biofilm infection and improved osseointegration. In addition, the prepared NiTi‐TiO_2‐x_ guidewire enables rapid thrombolysis (30 min) in the deep vein thrombosis of beagles after 10 min of sonothermal/urokinase treatment. The electronic chip‐mimetic sonothermal platform provides a promising and widespread clinical application prospect.

## Graphical abstract

Inspired by the thermal effect of working electronic chips, which consist of various heterostructures, an sonothermal‐responsive platform has been developed through constructing metal‐semiconductor interface on implants. Biofilm infection and deep vein thrombosis can be successfully treated by controllable ultrasound treatments.

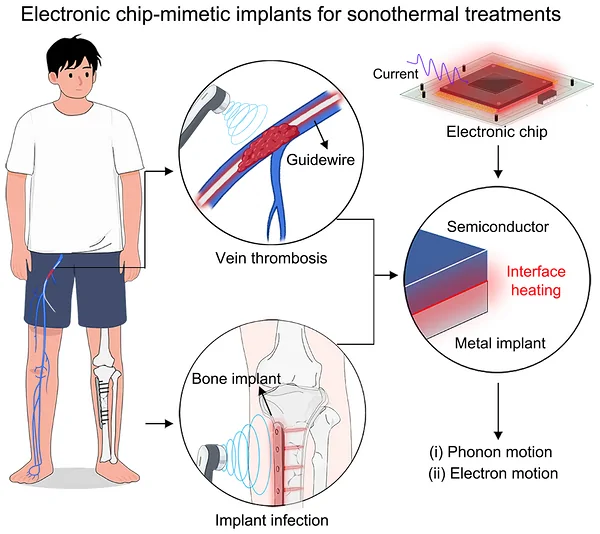

## 1 Introduction

Implantable medical devices play an important role in the repair and reconstruction of tissue functions, as well as in diseases treatments and monitoring [1]. The sterility and functional durability of the implants determine their lifespan and therapeutic efficacy. Once bacterial infection occurs on the surface of implants, it leads to implant loosening and damage to the surrounding tissue [2, 3]. Particularly when bacterial biofilms are formed on the implants surface, it is difficult for traditional antibiotic to eliminate the infection [4, 5]. In such cases, the implantation is considered to be a failure, often necessitating a second operation for the patient. Additionally, for biofunctional implants such as having drug‐delivery systems, the release process is typically uncontrollable, and the implants lose therapeutic effect once the drug is depleted [3]. For diseases monitoring, implanted devices require power supply to maintain their durable functionality, which usually involves complex circuit design and wireless transmission components. Therefore, the implantable medical devices should possess non‐invasive, long‐term, and controllable antibacterial and biological functions, which is essential for their long‐term clinical applications.

If an implant can generate in situ heat under external stimulation, it can be activated to exert specific biological effects. Photo‐thermal therapy and microwave‐thermal therapy can't meet the requirements due to the limited penetration of light and the unexplored microwave‐responsive implant. Ultrasound (US), with controllable, non‐invasive, and deeply penetrative properties, has been widely used in clinical imaging, rehabilitation therapy, tumor, and infection treatments [6, 7]. High‐intensity focused US (HIFU), for instance, can generate high temperatures in the deep tissues to destroy tumors [6]. However, HIFU is difficult to apply over large areas, and its high temperatures can cause tissue damage with prolonged exposure [8]. Thus, if low‐intensity US can selectively induce in situ heating of implants without damaging surrounding tissues, it would realize the external control of the implants' in vivo functions, such as localized antibacterial activity, drug release or other biological effects.

Therefore, the key to solving this problem lies in endowing implants with in situ sonothermal capability. Electronic devices such as computers and smartphones generate heat during prolonged working [9, 10, 11, 12, 13]. This is due to that the Joule heating can be produced by electronic chips when electric current passes through them. The primary reason for chips heating is the presence of various heterostructures, where electron and phonon transport through heterogeneous interfaces cause energy dissipate as heat [14]. Inspired by this, we hypothesize that constructing heterogeneous interfaces on the surface of implants may confer sonothermal effects, as US can excite phonon and electron motion in metals or semiconductors (Figure 1a,b).

**FIGURE 1:**
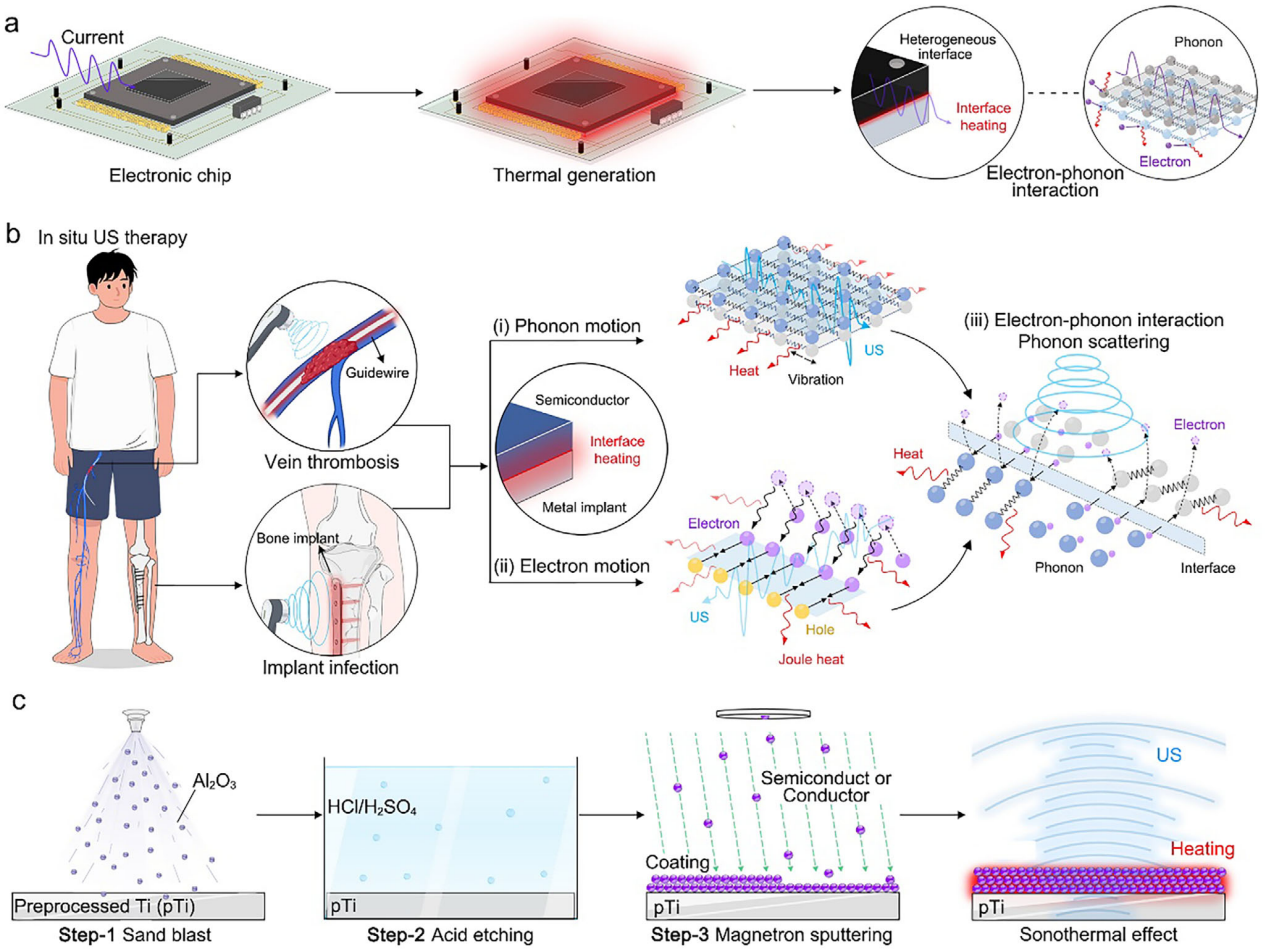
Schematic representation of the electronic chip‐mimetic sonothermal implants. (a) Joule heating can be produced by electronic chips when electric current passes through them. (b) Constructing heterogeneous interfaces on the surface of implants may confer sonothermal effects for the treatments of implant infection and vein thrombosis, as US can excite phonon and electron motion in metals or semiconductors (partly created in BioRender, DA29YPJQ4J). (c) Different semiconductor or conductor coatings were deposited on the titanium substrate surface via magnetron sputtering.

In this study, a series of conductor or semiconductor coatings were prepared on titanium substrates using magnetron sputtering, and their sonothermal effects were investigated under US irradiation (Figure 1c). We found that semiconductor coatings could impart different sonothermal effects to titanium, whereas metal coatings could not. The underlying sonothermal mechanism was summarized, which was related to the US‐induced electron and phonon motion. Finally, in an implant‐associated bone infection model, we prepared a titanium implant with defected TiO_2‐x_ coating, which showed a strong sonothermal ability. The combination of whitlockite (WH, (Ca_18_Mg_2_(HPO_4_)_2_(PO_4_)_12_)) on TiO_2‐x_ endowed the implant with sonothermal /sonodynamic effects. Through US treatment, the bacterial infection on the implant surface was eradicated and the osseointegration between implant and bone tissue was promoted via sono‐current. For the treatment of deep vein thrombosis of beagles, the prepared a NiTi‐TiO_2‐x_ guidewire combined with urokinase (UK) successfully dissolved the thrombosis under 10 min of US treatment.

## 2 Results

### 2.1 Sonothermal Effects of Conductor or Semiconductor Coatings on Titanium

Since the increase of interface roughness contributes to the bonding strength of coating and phonon scattering, prior to coating preparation on the titanium substrate, the titanium surface was treated by sandblasting and acid etching to achieve a certain roughness (pTi). Subsequently, different coatings were deposited on the titanium substrates' surface via magnetron sputtering (Figure 1c). These coatings included different conductors (tungsten (W), iron (Fe), molybdenum (Mo), or zinc (Zn)) and semiconductors (titanium dioxide (TiO_2_), silicon (Si), zinc oxide (ZnO), or tellurium (Te)) (Figure S1). The deposition of these coatings was motivated by the fact that some heterojunctions in electronic chips consist of metals and semiconductors. Subsequently, under US treatment (1 MHz, 1.2 W/cm^2^, continuous), the sonothermal temperatures of all samples were recorded. As shown in Figure 2a,b, after ten minutes of US irradiation, the temperatures of pTi, pTi‐W, pTi‐Fe, pTi‐Mo, and pTi‐Zn reached 23.4°C, 28.1°C, 28.4°C, 29.6°C, and 33.1°C, respectively. In contrast, the temperatures of pTi‐TiO_2_, pTi‐Si, pTi‐ZnO, and pTi‐Te reached 41.6°C, 44.4°C, 51.2°C, and 66.4°C, respectively. It was observed that the sonothermal effects of semiconductor coatings were generally stronger than those of conductor coatings. Notably, the Te coating reached 66.1°C after just one minute, exhibiting the highest thermal conversion efficiency. Therefore, the semiconductor coatings could universally endow titanium substrates with sonothermal effects, and the heating efficiency depended on the type of semiconductor.

**FIGURE 2:**
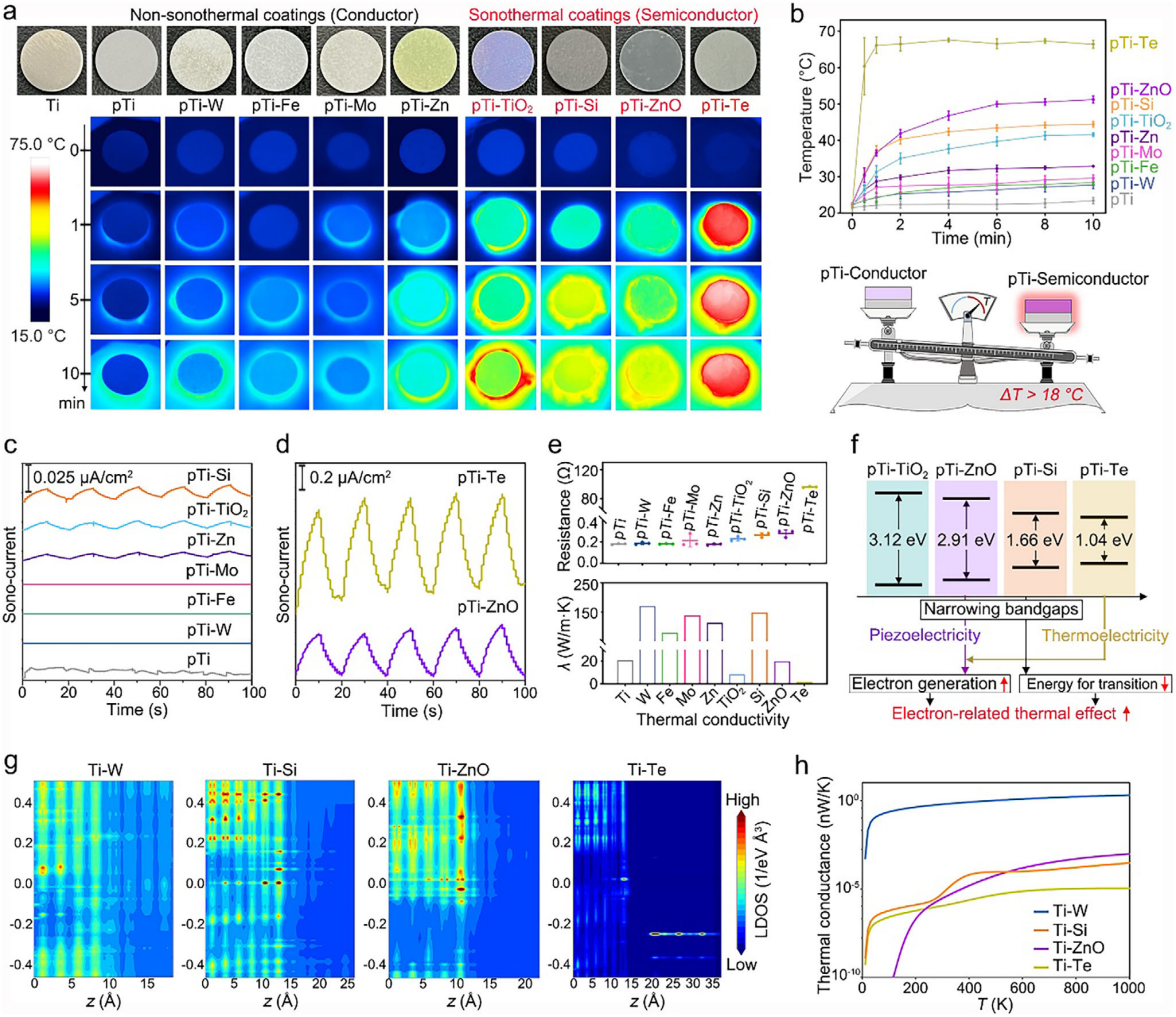
Sonothermal effect and electron‐related properties of all samples. (a) Images of Ti plates with different conductors or semiconductors and their thermal imaging under 10 min of US. (b) The corresponding heating curves of samples. n = 3 independent experiments per group, data are expressed as mean ± SD. (c, d) Current generated by the samples under US excitation. (e) Resistance of the samples and thermal conductivity of the coatings. n = 3 independent experiments per group, data are expressed as mean ± SD. (f) Bandgap of pTi‐semiconductors and the summarized factors for electron‐related thermal effect. (g) Local density of states and (h) the corresponding electronic thermal conductance of different pTi‐semiconductors.

### 2.2 Electron‐Related Sonothermal Mechanisms

Next, the heating mechanisms of these samples were investigated. Considering that the Joule heat can be generated when electrons pass through resistor, the current generated by the samples was detected under US. As shown in Figure 2c, no current signal was observed in the pTi‐W, pTi‐Fe, and pTi‐Mo groups, indicating that US did not excite free electrons from them. In contrast, pTi‐TiO_2_, and pTi‐Si exhibited obvious sono‐current, due to the US‐excited electrons in the semiconductor coatings. Notably, Ti‐ZnO and pTi‐Te showed a substantial increase in sono‐current (Figure 2d), which was in accordance with their heating trends. This might be attributed to the piezoelectric effect of ZnO [15] and the thermoelectric effect of Te [16], which generated significant current under US and thermal stimulation, respectively. The pTi and pTi‐Zn showed relatively weak sono‐current, possibly because oxide layers (TiO_2_ and ZnO) on the surfaces of pTi and pTi‐Zn. Since Joule heat from electron transport depends on current and resistance, then the resistance of the samples was measured. From Figure 2e, conductor‐coated samples exhibited lower resistance, whereas semiconductor‐coated samples had higher resistance, particularly for Te. Additionally, except for Si, semiconductors generally had lower thermal conductivity than conductor coatings [17, 18, 19], suggesting that low thermal conductivity was helpful in local heating due to the difficult heat conduction, and the thermal conductivity was not the only reason for sonothermal effect. Since US can excite electrons in semiconductors to transition from the valence band to the conduction band, the bandgap of a semiconductor coating was measured, which determines the energy barrier of electron excitation. A narrower bandgap means a lower energy barrier, facilitating the generation of more free electrons under US. As shown in Figure S2 and Figure 2f, pTi‐Te had the smallest bandgap. However, the bandgap sizes of different semiconductor coatings did not fully correlate with their heating trends, possibly because the US‐generated free electrons were also influenced by factors such as piezoelectric and thermoelectric effects.

To further explain these observations, the representative samples (Ti‐W, Ti‐Si, Ti‐ZnO, and Ti‐Te) were selected for density functional theory (DFT) calculations to investigate the electronic density of states (DOS) at the heterojunctions (Figure S3). As shown in Figure 2g, the results of local density of states (LDOS) showed that the electron were distributed on both side of Ti and W, indicating that the electrons were easy to transfer in the interface due to the small potential barrier between metals. In contrast, the electrons in the Si, ZnO, and Te sides were much less than W. The corresponding electronic thermal conductance of Ti‐Si, Ti‐ZnO, and Ti‐Te were also remarkably lower than that of Ti‐W (Figure 2h), which might be due to their higher potential barrier and the lower electrical conductivity of semiconductors compared with metals, and the electronic energy would transfer to the lattice atom and resulted in the higher thermal production.

### 2.3 Phonon‐Related Sonothermal Mechanisms

In addition to generating US‐induced electrons, as a mechanical wave, US can also induce phonon motion when propagating through a medium. Finite element analysis (FEM) was used to simulate the heat generation in Ti‐W, Ti‐Si, Ti‐ZnO, and Ti‐Te samples under US irradiation (Figure 3a). Under the same physical field and ultrasonic output power (1.2 W/cm^2^, 1 MHz), the samples showed different temperatures with the heating trend of Ti‐Te (351.29 ± 5.48 K) > Ti‐ZnO (323.63 ± 6.88 K) > Ti‐Si (318.45 ± 0.81 K) > Ti‐W (299.42 ± 0.53 K), which was similar to the experimental results. The sonothermal performance was related to the physical parameters of coatings, mainly including sound velocity, mass density, thermal conductivity, and specific heat capacity. Among these parameters, the thermal conductivity and specific heat capacity determined the thermal conduction of samples. The thermal conductivity of W, Si, ZnO, and Te were 170, 148, 20, and 2 W/m·K, respectively, indicating that low thermal conductivity contributed to their sonothermal effect. The specific heat capacity of W, Si, ZnO, and Te were 130, 700, 495, and 200 J/kg·K, respectively. Except for W, low specific heat capacity was generally beneficial for sonothermal effect.

**FIGURE 3:**
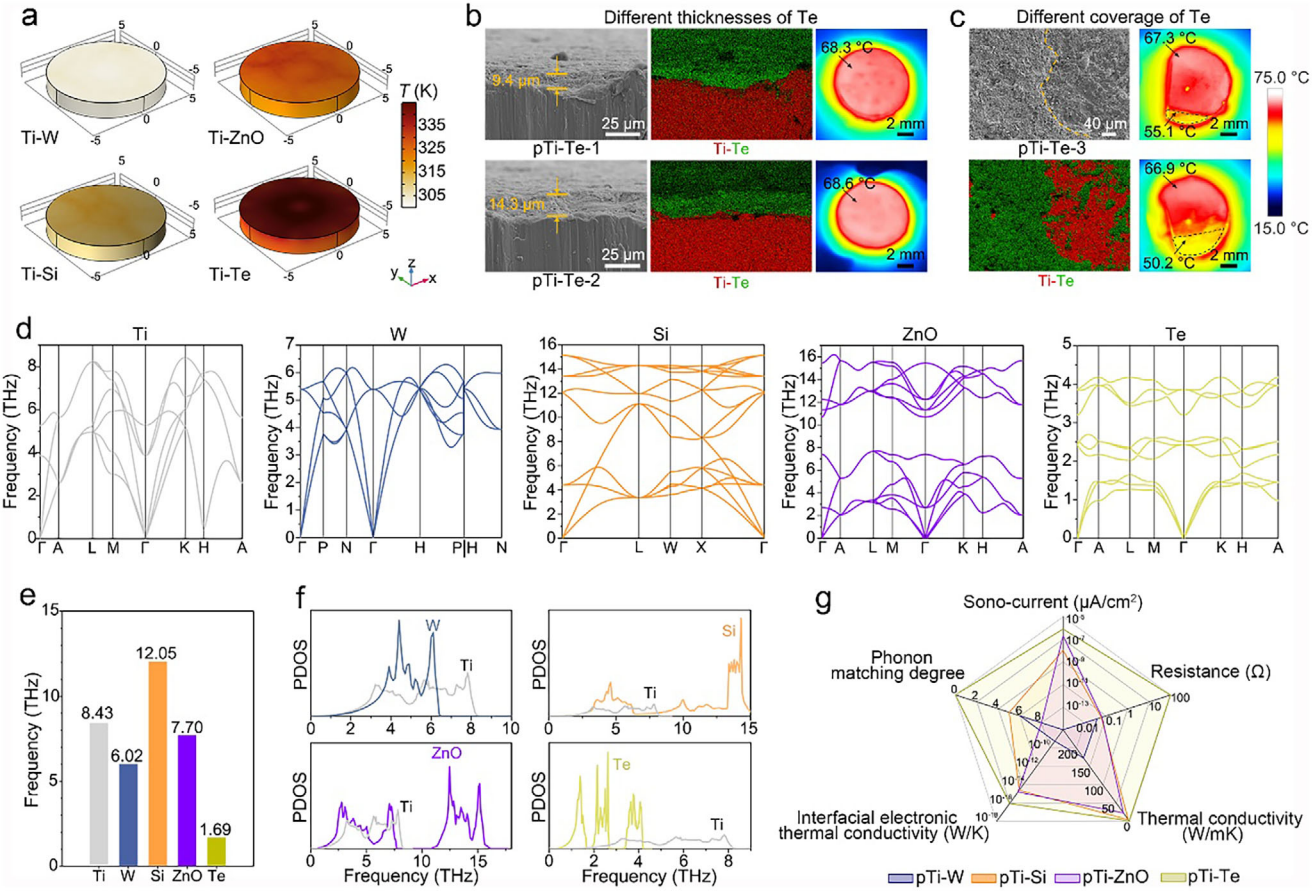
Phonon‐related sonothermal mechanisms. (a) Finite element analysis of the heat generation in Ti‐W, Ti‐Si, Ti‐ZnO, and Ti‐Te samples under US irradiation. (b) Different thickness and (c) coverage of Te coating on pTi and the corresponding thermal imaging. (d) Phonon dispersion relations, (e) cutoff frequencies, and (f) phonon density of states of Ti, W, Si, ZnO, and Te. (g) Radar map summarized the influence factors of the implants' sonothermal effect, including sono‐current, resistance, thermal conductivity, interfacial electronic thermal conductivity, and phonon matching degree.

To investigate the influence of coating's thickness and coverage on sonothermal effect, different thickness (Figure 3b) and coverage (Figure 3c) of Te coating on pTi were prepared. The thermal imaging results showed that Te coatings with 9.4 µm and 14.3 µm had similar sonothermal effect, while lower coverage led to reduced temperature, indicating that the area of heterogeneous interface might play a leading role on the sonothermal effect.

Furtherly, the phonon dispersion relations and phonon density of states (PDOS) of Ti, W, ZnO, Si, and Te were calculated using density functional theory (DFT) [20] (Figure S4). As shown in Figure 3d,e, W (6.02 THz) and Ti (8.43 THz) exhibited similar cutoff frequencies in their acoustic branches, whereas Si (12.05 THz) and Te (1.69 THz) showed obvious differences in acoustic cutoff frequencies compared with Ti, leading to poor phonon group velocity matching. Notably, Te's optical branch group velocity approached zero, while Ti's acoustic branch group velocity was relatively high. The close frequency overlap between them resulted in a strong coupling, thereby impeding heat flow. The PDOS results further demonstrated that the overlap between Si, ZnO, Te, and Ti were much weaker than that between Ti and W. Since the optical‐phonon frequencies of ZnO were much higher than the acoustic branches of Ti, the energy of its high‐frequency optical phonons cannot be transferred directly to Ti's low‐frequency acoustic branches via phonon–phonon scattering, resulting in a reduced interfacial thermal conductance (Figure 3f). The mismatches in phonon dispersion, phonon group velocity (Figure S5), and PDOS caused the US wave to encounter significant interfacial resistance during propagation, leading to excessive reflection and scattering [21]. Ultimately, this energy is dissipated as heat. Taken together, the influence factors of the implants' sonothermal effect were summarized in a radar map, including sono‐current, resistance, thermal conductivity, and phonon matching degree (Figure 3g). We found that high sono‐current and resistance, combined with low thermal conductivity and low phonon matching degree of Ti and coatings, contributed to the sonothermal performance of Ti‐based implants.

### 2.4 Sonothermal‐Responsive pTi‐TiO_2‐x_‐WH Implant for Anti‐Bacteria

After elucidating the sonothermal properties of semiconductor coatings, we sought to select a suitable titanium‐based implant for biomedical application, such as treating bone implant‐related infection. Among these coatings, Te had the strongest sonothermal effect. However, its biocompatibility has not been clinically validated, posing potential biosafety risks and hindering its future clinical translation. Since pTi‐TiO_2_ had moderate sonothermal effect and clinically used titanium implants inherently feature a surface oxide layer (TiO_2_), we focused on processing the oxide layer on pTi for better US‐responsive practical applications. Given that structural defects in materials can enhance phonon and electron scattering [22] to reduce thermal conductivity, the pTi was treated by high‐temperature sulfurization treatment to introduce defect structure (pTi‐TiO_2‐x_). Since the mild thermal effect cannot kill bacteria [23], and the osseointegration performance of titanium implants also needs to be improved, the WH coatings were further prepared on the surface of pTi‐TiO_2‐x_ through high‐temperature calcination (Figure 4a). As WH is a natural bone component and has piezoelectric properties [24, 25, 26], it was expected to achieve rapid biofilm elimination and promote subsequent osseointegration of implants through US treatments. Intriguingly, compared with magnetron sputtered pTi‐TiO_2_ (41.6°C) and pTi (23.4°C), both sulfurized pTi‐TiO_2‐x_ (50.6°C) and pTi‐TiO_2‐x_‐WH (50.9°C) exhibited stronger sonothermal performance under 10 min of US irradiation (Figure 4b and Figure S6). As shown in Figure 4c, the TEM images of pTi‐TiO_2‐x_ showed that the lattice planes of (110) and (111) belong to rutile TiO_2_. The lattice distortion confirmed the presence of defect structure, which was developed by the sulfur‐induced oxygen vacancy in TiO_2_. From Figure 4d, pTi‐TiO_2‐x_ exhibited remarkably enhanced sono‐current intensity compared with pTi‐TiO_2_, which might be due to that the introduction of defect level improved the generation of US‐excited electron. The greatly increased resistance of pTi‐TiO_2‐x_ was likely because of the doping of insulating sulfur (Figure 4e). The bandgap of pTi‐TiO_2‐x_ was also reduced after the introduction of defect structure (Figure S7a and Figure 4f), which also contributed to the generation of sono‐current. These results were in accordance with our summarized determinants of sonothermal effect, and also validated our hypothesis that defect engineering could enhance the sonothermal effect of TiO_2_ coating. XRD results showed that the TiO_2‐x_ in pTi‐TiO_2‐x_ is rutile phase, and the appearance of the (0 2 10) and (2 2 0) peaks was attributed to the WH in pTi‐TiO_2‐x_‐WH (Figure 4g) [27]. SEM results showed that WH nanoparticles were densely and evenly distributed on the surface of pTi‐TiO_2‐x_‐WH. In addition, cross‐sectional images showed the uniform distribution of Mg, Ca, and P elements in the coating, which confirms the successful preparation of the WH coating (Figure 4h). X‐ray photoelectron spectroscopy (XPS) analysis revealed that pTi‐TiO_2‐x_‐WH exhibited additional Mg 1s, Ca 2p, and P 2p peaks compared with pTi‐TiO_2‐x_, further proving the modification of WH (Figure S8). Nanoindentation results showed that the coating on pTi‐TiO_2‐x_ was very stable (elastic modulus = 180.6 GPa), and the elastic modulus of the coating decreased to 16.3 GPa after the introduction of WH, but it still had sufficient stability and could match the elastic modulus of bone tissue (Figure S7b). Since WH is a natural piezoelectric material, the piezoelectric property of pTi‐TiO_2‐x_‐WH was studied by piezoresponse force microscopy (PFM). As shown in Figure 4i, the phase voltage signal of pTi‐TiO_2‐x_‐WH showed a 180° phase shift and a typical butterfly amplitude curve, with a maximum amplitude of 7 nm, and its piezoelectric coefficient d_33_ reached 699.9 pm/V. Compared with pTi‐WH (d_33_ = 217.6), the piezoelectric property of pTi‐TiO_2‐x_‐WH had been greatly improved (Figures S9 and S10), which might be due to the formation of a heterojunction between TiO_2‐x_ and WH, which promoted the polarization of WH. Through electron spin resonance detection (Figure 4j), compared with other groups, pTi‐TiO_2‐x_‐WH generated more hydroxyl radicals under US due to the polarization of WH on surface, indicating that the introduction of WH enhanced the sonodynamic effect of the titanium substrate. Based on the sonothermal/sonodynamic effects of pTi‐TiO_2‐x_‐WH, its anti‐biofilm performance against MRSA was investigated. From the results of the spread plate experiment, the antibacterial efficiencies of the pTi‐TiO_2‐x_ (15 min), pTi‐TiO_2‐x_‐WH (15 min), and pTi‐TiO_2‐x_‐WH (15 min, 2 cycles) groups were calculated to be 72.8%, 91.4%, and 99.2%, respectively (Figure 4k). During the ultrasonic process, the temperature of the samples never exceeded 50°C, ensuring a safe US treatment. The results of the bacterial live/dead staining were consistent with the trends of spread plate experiment, with almost no live bacteria observed in the pTi‐TiO_2‐x_‐WH (15 min, 2 cycles) group (Figure 4l).

**FIGURE 4:**
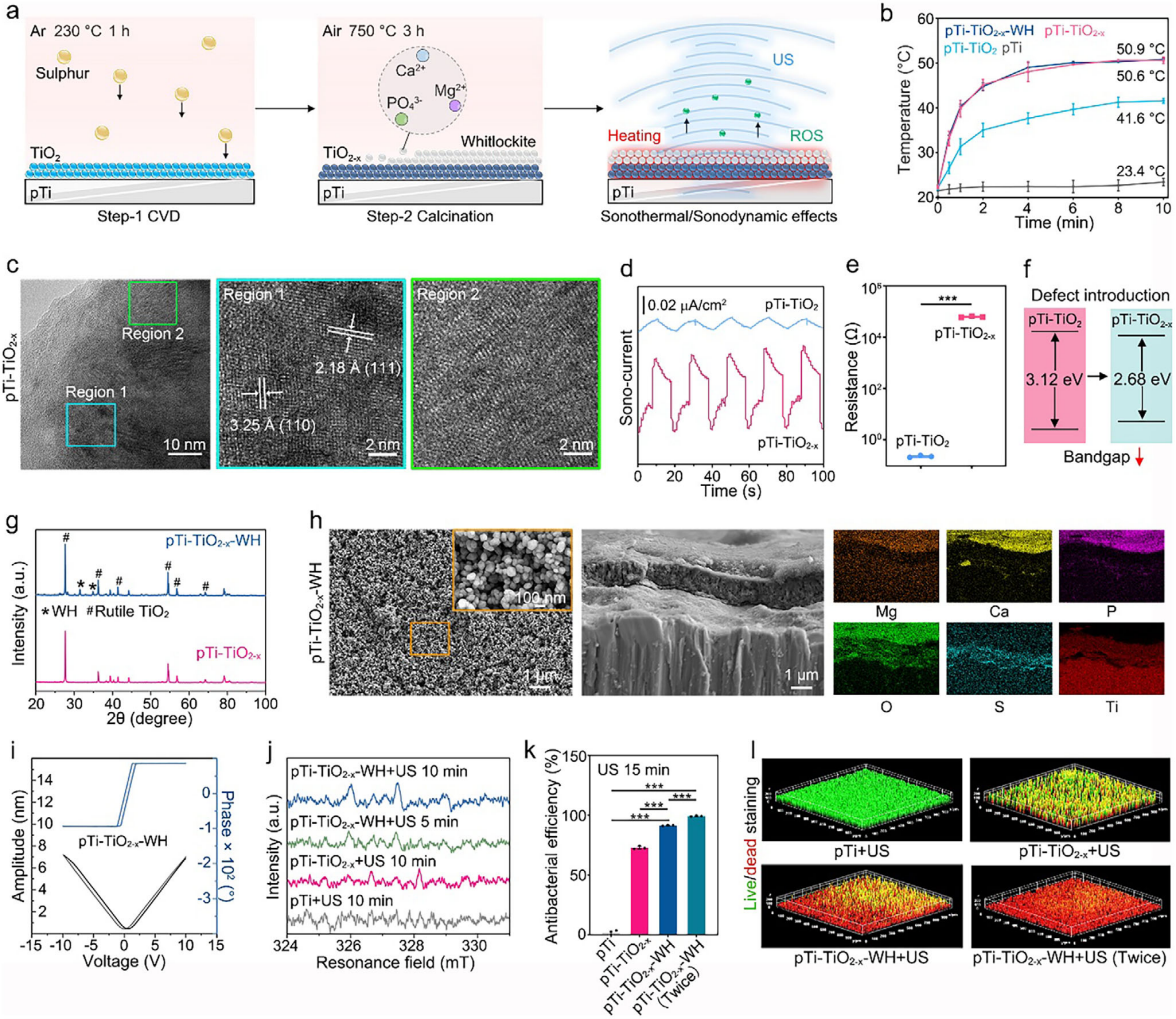
Sonothermal‐responsive pTi‐TiO_2‐x_‐WH implant for anti‐bacteria. (a) Preparation process of pTi‐TiO_2‐x_‐WH. (b) Sonothermal performance of pTi, pTi‐TiO_2_, pTi‐TiO_2‐x_, and pTi‐TiO_2‐x_‐WH. n = 3 independent experiments per group, data are expressed as mean ± SD. (c) TEM measurements of pTi‐TiO_2‐x_. (d) Current generated by pTi‐TiO_2_ and pTi‐TiO_2‐x_ under US excitation. (e) Resistance and (f) bandgap of pTi‐TiO_2_ and pTi‐TiO_2‐x_. (g) XRD of pTi‐TiO_2‐x_‐WH. (h) SEM of pTi‐TiO_2‐x_‐WH and its energy dispersive spectrum (EDS) mapping. (i) PFM test of pTi‐TiO_2‐x_‐WH. (j) Hydroxyl radicals generated by pTi+US, pTi‐TiO_2‐x_+US, and pTi‐TiO_2‐x_‐WH+US. (k) Antibacterial efficiency and (l) live/dead staining of pTi+US, pTi‐TiO_2‐x_+US, and pTi‐TiO_2‐x_‐WH+US groups. n = 3 independent experiments per group, data are expressed as mean ± SD. ^***^ *p* < 0.001, one‐way ANOVA.

### 2.5 Osteogenic Activity In Vitro

In addition to potent antibacterial properties, orthopedic implants must also possess the ability to promote osseointegration, which helps to accelerate patients' recovery. The cell viability of human bone marrow mesenchymal stem cells (hBMSCs) on samples was evaluated by CCK8 test. From Figure S11, after 1, 3, and 7 days of co‐culture, the pTi, pTi‐TiO_2‐x,_ and pTi‐TiO_2‐x_‐WH showed no obvious cytotoxicity. Although the cell viability in pTi‐TiO_2‐x_‐WH+US group was relatively reduced, it gradually increased on Day 3 and Day 7, indicating that the short US treatment did not influence the subsequent cell growth. Since WH is one of the components of natural bone and its piezoelectric effect is conducive to bone regeneration, the osteogenic properties of pTi‐TiO_2‐x_‐WH and pTi‐TiO_2‐x_‐WH+US on hBMSCs were tested using qRT‐PCR (Figure 5a). After 14 days of co‐culture of the samples with hBMSCs, the expression of osteogenesis‐related genes *ALP*, *Runx2*, *Coll I*, *OPN*, and *OCN* were enhanced in both of the pTi‐TiO_2‐x_‐WH and pTi‐TiO_2‐x_‐WH+US groups, while the osteogenic gene expression in the pTi‐TiO_2‐x_‐WH+US group generally higher than that of the pTi‐TiO_2‐x_‐WH group. This indicated that WH with Ca and Mg components could promote the osteogenesis of MSCs, and its US‐piezoelectric effect further enhanced the expression of osteogenic genes [24]. Alizarin red staining results showed that pTi‐TiO_2‐x_‐WH+US had the best performance to promote the mineralization of hBMSCs (Figure 5b). The expression of osteogenic proteins OPN and Coll I was detected by immunofluorescence staining, and the results showed that the cells in all groups had normal morphology, indicating good biocompatibility of the samples. The fluorescence expression of OPN and Coll I was strongest in the pTi‐TiO_2‐x_‐WH+US group, further confirming its excellent osteogenic properties (Figure 5c,d). The RNA sequencing results showed that compared with the pTi group, the pTi‐TiO_2‐x_‐WH+US group had 1143 upregulated genes and 562 downregulated genes (Figure 5e and Figure S12a). The enriched gene ontology (GO) terms related to positive regulation of ossification, bone mineralization, mechanical stimulus, bone morphogenesis, positive regulation of MAPK cascade were up‐regulated in pTi‐TiO_2‐x_‐WH+US group (Figure 5f). The enriched Kyoto encyclopedia of genes and genomes (KEGG) results showed that the Calcium signaling pathway and Pl3K‐Akt signaling pathway were significantly up‐regulated by piezoelectric effect [28, 29] (Figure 5g). Gene set enrichment analysis showed that gap junction, mechanosensitive monoatomic ion channel activity, positive regulation of calcineurin mediated signaling, and osteoblast signaling were upregulated (Figure S12b). The heatmap exhibited that the genes of osteogenic, calcium influx, angiogenesis, and energy supply were up‐regulated due to the US‐induced electrical signal [30, 31, 32], which could improve the osteogenic differentiation of hBMSCs (Figure 5h).

**FIGURE 5:**
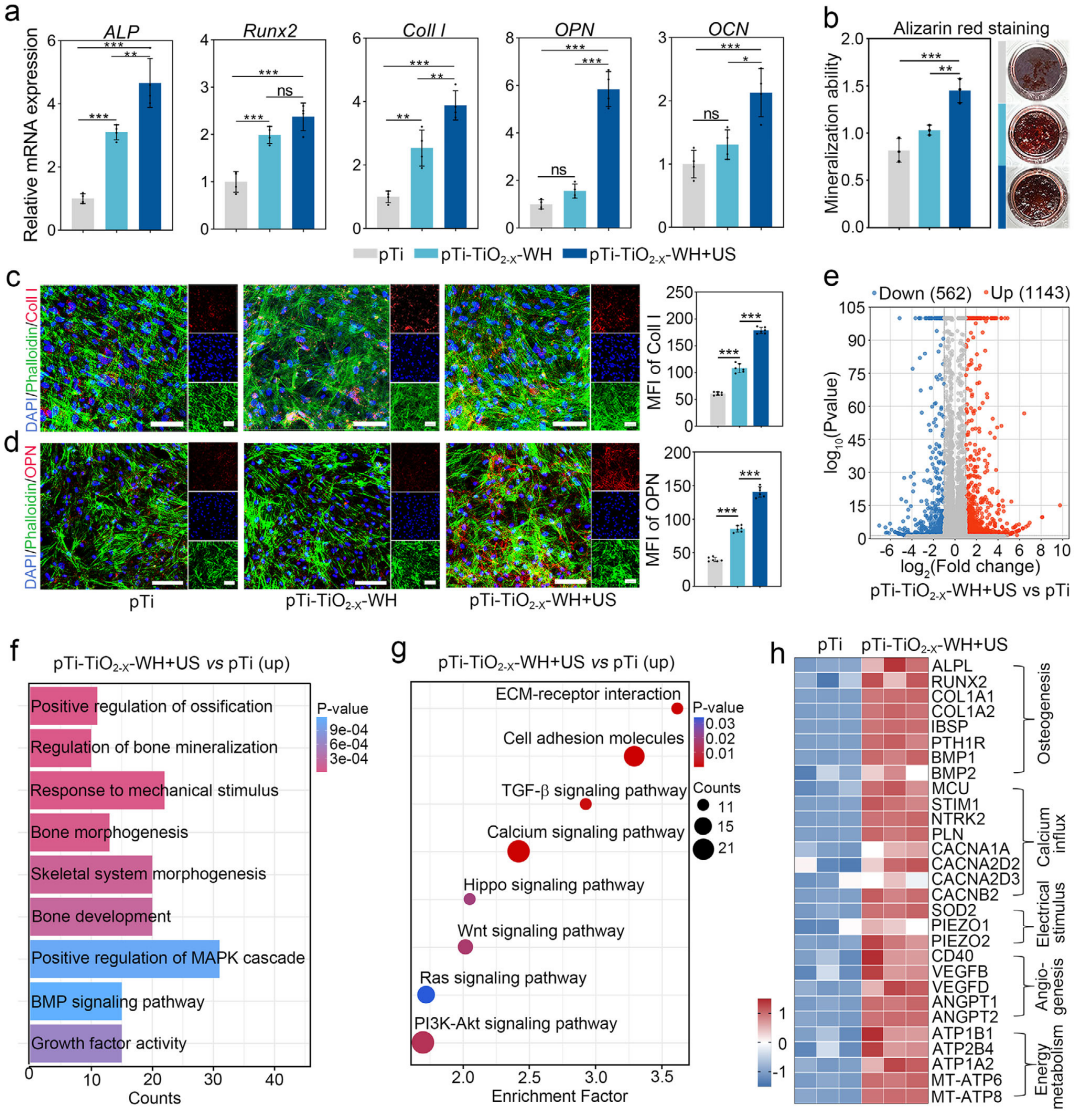
Osteogenic activity in vitro. (a) Osteogenesis‐related genes expression of *ALP*, *Runx2*, *Coll I*, *OPN*, and *OCN*. n = 4 independent experiments per group, data are expressed as mean ± SD. ^*^ *p* < 0.05, ^**^ *p* < 0.01, ^***^ *p* < 0.001, one‐way ANOVA. (b) Alizarin red staining. n = 3 independent experiments per group, data are expressed as mean ± SD. ^**^ *p* < 0.01, ^***^ *p* < 0.001, one‐way ANOVA. (c, d) Immunofluorescence staining of Coll‐I and OPN. n = 6 independent experiments per group, data are expressed as mean ± SD. ^***^ *p* < 0.001, one‐way ANOVA. Scale bar = 100 µm. (e) Volcano plots of pTi‐TiO_2‐x_‐WH+US vs pTi. Genes with adjusted *p* < 0.05 are shown in red or blue dots (n = 3 independent experiments per group). (f, g) GO and KEGG enrichment analysis for up‐regulated genes in pTi‐TiO_2‐x_‐WH+US. (h) Genes associated with osteogenic, calcium influx, angiogenesis, and energy supply (n = 3 independent experiments per group).

### 2.6 Antibacterial and Osteogenic Activities In Vivo

After investigating the in vitro antibacterial and osteogenic properties of the implants, the in vivo therapeutic effects were studied. Before implantation, the MRSA biofilms were cultured on the surface of the implants, followed by implanting the samples into the bone tissue of rats. Post bone infection, the implant sites were irradiated with US (1.2 W/cm^2^, 1 MHz, 15 min, 2 cycles for anti‐biofilm; 0.3 W/cm^2^, 1 MHz, 15 min, per 2 days for osteogenesis), and subsequently, the assessments for antibacterial and osteogenic performance were carried out. Besides, the in vivo osteogenic performance of uninfected titanium implants was also evaluated (Figure 6a). As shown in Figure 6b, after surface modification, the coating distribution on the bone screw surface was uniform, demonstrating the feasibility of our modification strategy for clinical bone implants. The bone screws were then implanted into the tibial plateau of rats. Subsequently, the heat generation of implants at the implanted sites was monitored using a thermal imager. From Figure 6c, after 15 min of US irradiation, the surface temperature of pTi only reached 35.1°C. In contrast, the surface temperature of pTi‐TiO_2‐x_‐WH increased to 50.7°C, proving that its sonothermal effect was also applicable in vivo.

**FIGURE 6:**
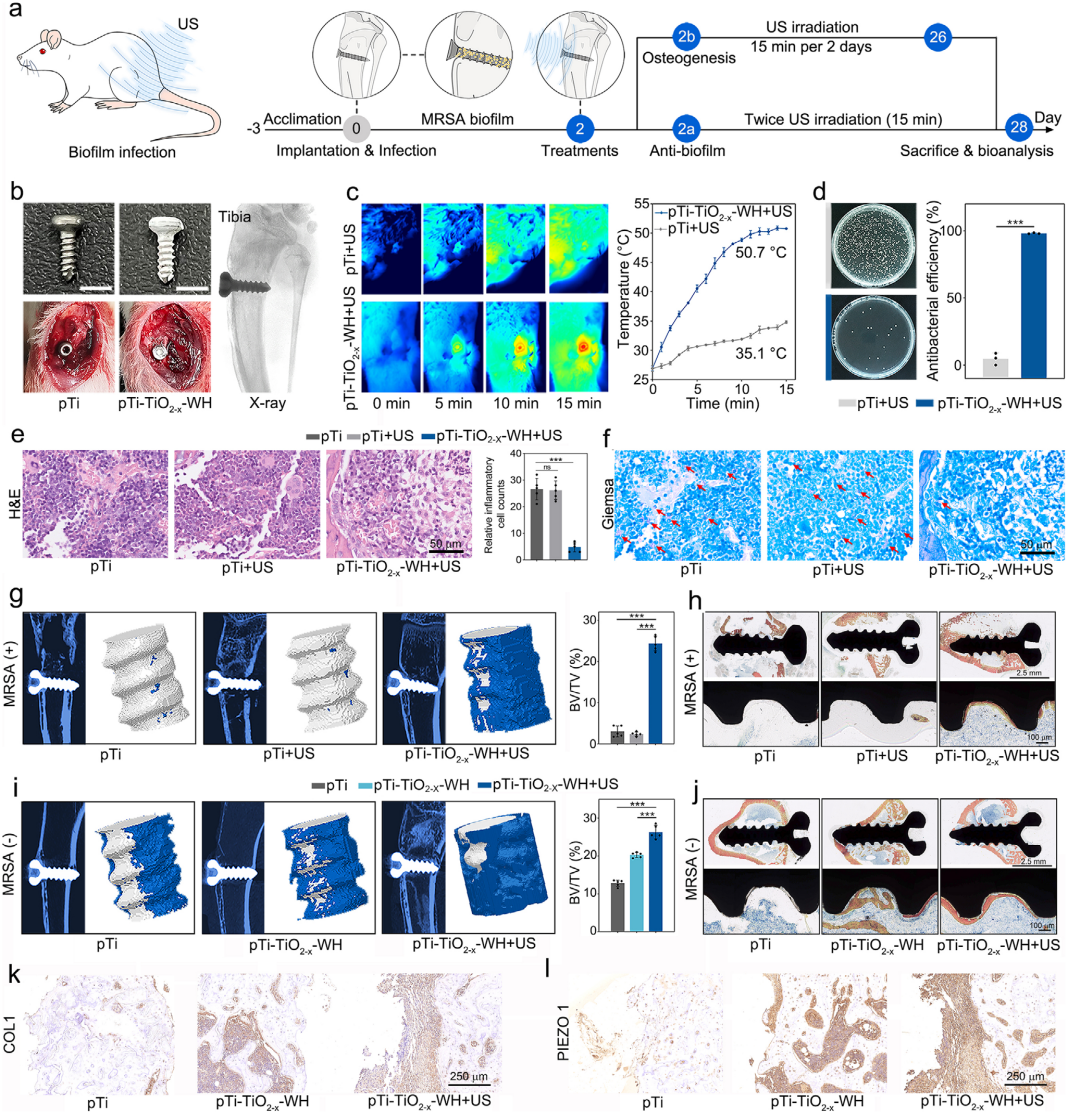
Antibacterial and osteogenic activities in vivo with or without MRSA biofilm infection. (a) Flowchart of animal experiments. (b) Images of pTi and pTi‐TiO_2‐x_‐WH bone screws and the implanted site of rats. Scale bar = 3 mm. (c) Thermal imaging and related heating curves of pTi and pTi‐TiO_2‐x_‐WH bone screw in vivo under US irradiation. n = 3 independent experiments per group, data are expressed as mean ± SD. (d) In vivo antibacterial efficiency of pTi‐TiO_2‐x_‐WH against MRSA biofilms. n = 3 independent experiments per group, data are expressed as mean ± SD. ^***^ *p* < 0.001, one‐way ANOVA. (e, f) H&E and Giemsa staining of bone tissues around the implants, (g, i) Micro‐CT of bone tissues with implants and corresponding bone volume/total volume (BV/TV) analysis. n = 5 independent experiments per group, data are expressed as mean ± SD. ^***^ *p* < 0.001, one‐way ANOVA. (h, j) Van Gieson's picrofuchsin staining of the bone tissues on the surface of implants. (k, l) Immunohistochemical staining of COL I and PIEZO 1.

In the MRSA biofilm infected model, the bacteria on the implant surface after US treatment were collected for spread plate test. As shown in Figure 6d, the antibacterial efficiency of pTi‐TiO_2‐x_‐WH against MRSA biofilms reached 99.0%. After 28 days, the H&E and Giemsa staining of bone tissues around the implants showed that the number of inflammatory cell and bacteria in the pTi‐TiO_2‐x_‐WH+US group was obviously less than that of the pTi and pTi+US groups, indicating that the inflammatory reaction was relieved due to the elimination of biofilm by US therapy (Figure 6e,f). The bone integration on the bone screw surface was assessed using micro‐CT. From Figure 6g,i, in the infected model, the bone mass on the surface of the pTi‐TiO_2‐x_‐WH+US group was significantly higher than that of the other groups. In the uninfected model, the bone mass on the surface of pTi‐TiO_2‐x_‐WH and pTi‐TiO_2‐x_‐WH+US was higher than that of the pTi group, while the pTi‐TiO_2‐x_‐WH+US group showed the best osseointegration, consistent with the results of cell experiments. The newly formed bone tissues on the surface of implants were then stained by Van Gieson's picro fuchsin. As shown in Figure 6h,j, in both the infected and uninfected models, the pTi‐TiO_2‐x_‐WH+US surface exhibited the highest amount of new bone formation. The immunohistochemical staining of Collagen type I (Coll I) and Piezo1 was performed at the bone defect site following screw removal (Figure 6k,l). The results demonstrated that Coll I expression was markedly elevated in the pTi‐TiO_2‐x_‐WH+US group compared with both pTi‐TiO_2‐x_‐WH and pTi groups, indicating that WH promoted osteogenesis in vivo, with further enhancement upon US intervention. Piezo1 expression was significantly upregulated in the pTi‐TiO_2‐x_‐WH+US group, while pTi and pTi‐TiO_2‐x_ groups had no statistically significant difference. These findings indicated that US stimulation was essential for Piezo1 activation, ultimately promoting osteogenic differentiation and bone repair at the defect site (Figure S13). No sign of thermal necrosis, osteolysis, or excessive inflammatory infiltration was observed from the H&E and Masson staining of pTi‐TiO_2‐x_‐WH+US group, indicating the good safety of sonothermal/piezoelectric treatment. The H&E staining of the main organs of rats showed that the implantation of pTi‐TiO_2‐x_‐WH did not exhibited organ toxicity (Figure S14). These results demonstrated that pTi‐TiO_2‐x_‐WH+US not only efficiently eliminated bacterial biofilm, but also promoted osseointegration through US treatments.

### 2.7 Thrombolysis Performance of NiTi‐TiO_2‐x_ Through Sonothermal Treatment

Since NiTi guidewire is widely used in cardiovascular intervention, the NiTi was treated by high‐temperature sulfurization treatment to obtain NiTi‐TiO_2‐x_. The sonothermal effect of NiTi and NiTi‐TiO_2‐x_ guidewires was measured by thermal imager under US irradiation. From Figure 7a, compared with NiTi (23.0°C), the NiTi‐TiO_2‐x_ guidewire (42.5°C) exhibited stronger sonothermal performance under 1 min of US irradiation, demonstrating that high‐temperature sulfurization also could endow NiTi with excellent sonothermal ability. In vitro thrombolysis assay, the blood clots were treated with PBS, PBS+US, UK, UK+US, NiTi‐TiO_2‐x_+US, and NiTi‐TiO_2‐x_+UK+US, respectively. The US irradiation (below 44°C) and co‐culture time were 10 min and 30 min, respectively. From Figure 7b–d, the thrombolytic rate and absorbance of fibrin in the supernatant of the above groups showed that UK had a weak thrombolytic ability during 30 min of treatment due to the slow enzymatic activity of UK. With the help of US, the thrombolytic rate was increased due to the assistance of the cavitation effect. Notably, the NiTi‐TiO_2‐x_+UK+US almost dissolved all of the blood clots, indicating that the sonothermal/UK treatment had an excellent synergistic thrombolytic effect.

**FIGURE 7:**
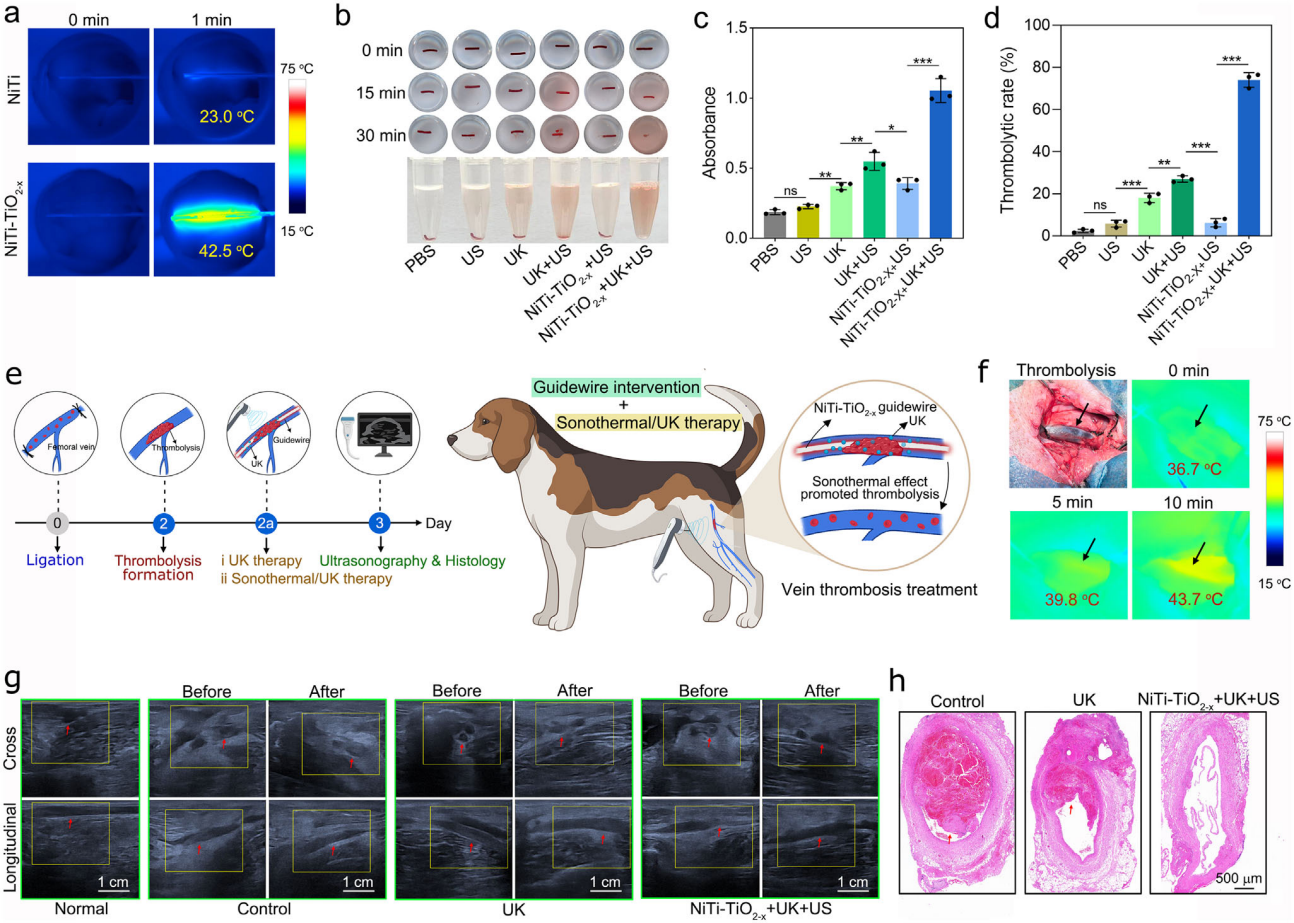
Thrombolysis performance of NiTi‐TiO_2‐x_ through sonothermal treatment. (a) Thermal imaging of NiTi and NiTi‐TiO_2‐x_ under 1 min of US irradiation. (b) In vitro thrombolysis assay of PBS, PBS+US, UK, UK+US, NiTi‐TiO_2‐x_+US, and NiTi‐TiO_2‐x_+UK+US. (c) Absorbance of supernatant at 450 nm (fibrin) (and d) corresponding thrombolytic rate of above groups. n = 6 independent experiments per group, data are expressed as mean ± SD. ^*^ *p* < 0.05, ^**^ *p* < 0.01, ^***^ *p* < 0.001, one‐way ANOVA. (e) Flowchart of thrombolysis experiments in vivo (partly created in BioRender, DA29YPJQ4J). (f) Image of femoral vein thrombus and the thermal imaging of thrombus after NiTi‐TiO_2‐x_+US treatment. (g, h) Ultrasound imaging and H&E staining of the veins after different treatments (the thrombus was indicated by red arrow).

To investigate the thrombolytic effect of NiTi‐TiO_2‐x_ in vivo, the femoral vein of beagles was ligated for 2 days to form a deep vein thrombus. After that, the beagles were treated by UK and NiTi‐TiO_2‐x_+UK+US (10 min), respectively (Figure 7e). Figure 7f showed the femoral vein thrombus and the thermal imaging of thrombus after sonothermal treatment (below 44°C), which proved that the sonothermal effect of NiTi‐TiO_2‐x_ guidewire was also applicable in vivo. The ultrasound imaging results showed that the thrombus was developed in each group before treatment. From cross and longitudinal sections, it was found that the thrombus (indicated by red arrow) still existed after UK treatment (30 min), while the thrombus signal almost disappeared in the NiTi‐TiO_2‐x_+UK+US group (Figure 7g). The corresponding H&E staining of the veins after different treatments also showed that successful clearance of thrombus in the NiTi‐TiO_2‐x_+UK+US group (Figure 7h). The above results demonstrated that 10 min of sonothermal/UK therapy had an excellent thrombolytic ability in vivo, which was obviously superior to the clinical UK therapy.

## 3 Discussion

In this study, inspired by the heat generation phenomenon in electronic chips, a series of conductors or semiconductors coatings were prepared on the surface of titanium implants using the magnetron sputtering method or high‐temperature sulfuration. By testing the sonothermal effect of the implants, we found that semiconductor coatings generally exhibited enhanced sonothermal effects, whereas conductor coatings did not. The sonothermal mechanism of semiconductor coatings was investigated, and we found that the sonothermal effect was closely related to the motion of electrons and phonons excited by US within the heterogeneous interface of implants. The sonothermal efficiency of metal‐semiconductor was closely related to the type of heterojunction and the coverage of the heterojunction interface. We found that the sonoelectric effect of semiconductor coatings was associated with sono‐current, bandgap, electrical resistance, and the type of semiconductor. In terms of phonon transport, heat conduction between metal and semiconductor was related to the phonon matching degree. Additionally, the structural defects in semiconductors also influenced the phonon‐electron interactions at the interface and played an important role in interfacial heat conduction. The prepared pTi‐TiO_2‐x_‐WH with excellent sonothermal effects, for bacterial biofilm infections in bone tissue of implants in vivo, achieved efficient and non‐invasive treatment through in situ sonothermal/sonodynamic effects.

In future clinical applications, once a patient experiences bacterial biofilm infection after surgical implantation, there will be no need for a secondary revision surgery; the infection can be eliminated simply through external US therapy. Compared with existing clinical treatment modalities, the core advantages of this strategy are as follows: (1) in contrast to systemic antibiotic administration, our system employed physical antimicrobial mechanisms to circumvent the development of drug resistance, while also eliminating visceral toxicity and substantially shortening the treatment duration; (2) deep tissue penetration was achieved through US‐driven activation, whereas local irrigation of infected sites can only cleanse superficial tissues and fails to penetrate into the deep regions of infected bone; (3) compared with the Ilizarov technique, our system integrated both antibacterial and osteogenic functions, significantly reducing treatment duration, eliminating the need for external fixation, and thereby preventing pin tract infections and associated complications, while also alleviating psychological burden on patients; (4) the Masquelet technique generally requires two or more surgical stages, while the pTi‐TiO_2‐x_‐WH+US system offered a non‐invasive approach with potent antibacterial efficacy, low toxicity, shortened treatment duration, high patient compliance. For the treatment of deep vein thrombosis in big animal model, we demonstrated the excellent thrombolytic ability of NiTi‐TiO_2‐x_ under sonothermal/UK treatment, which was notably superior to the UK treatment, offering a new strategy for rapid clinical thrombolysis. In addition to great sonothermal performance, the criteria for choosing semiconductors coatings should include: (1) good biocompatibility; (2) the degradation products of the semiconductor have certain biological functions, such as promoting osteogenesis and angiogenesis, inhibiting bacterial infection, and modulating immunity; (3) strong bonding strength between coating and implant. In the future, the sonothermal platform has potential be applied to diverse pathological conditions such as solid tumors, neurological disorders, and cardiovascular diseases.

## 4 Experimental Section

### 4.1 Chemicals

Calcium hydroxide (≥ 99%), magnesium hydroxide (≥ 99%), phosphoric acid (≥ 99%), dermaline sulfur (≥ 99%), and phosphorus pentoxide (≥ 99%) were purchased from Aladdin (Shanghai, China). The clinical bone screws (diameter: 1.5 mm; length: 6 mm) were purchased from Wego Medical Device (Shandong, China). Sputtering targets include zinc (Zn), molybdenum (Mo), tungsten (W), Iron (Fe), silicon (Si), zinc oxide (ZnO), tellurium (Te), and titanium dioxide (TiO_2_) were purchased from Zhongnuo Advanced Material (Beijing, China).

### 4.2 Preparation of Whitlockite (Ca_18_Mg_2_(HPO_4_)_2_(PO_4_)_12_) Nanoparticles

Calcium hydroxide (0.37 M) and magnesium hydroxide (0.13 M) were added into the deionized water (DI). The mixture was heated at 85°C and stirred for 60 min. After adjusting the pH to 5, phosphoric acid (100 mL, 0.5 M) was slowly added to this mixture over 8 min. Subsequently, the mixture was further heated to 100°C and stirred for 10 h. After that, the mixture was aged at 80°C for 14 h. The supernatant was removed after aging, and the solid was washed three times with DI. The solid was vacuum‐dried overnight to yield the final product (WH) [26].

### 4.3 The Preparation of pTi‐TiO_2‐x_‐WH

All pure titanium plates (Ф 33.9 mm, Ф 14.75 mm, Ф 8.0 mm, 1.4 mm in thickness) were polished to achieve surface treatment first. Titanium plates were sandblasted with 60‐mesh white corundum and etched with a sulfuric‐hydrochloric acid mixture at 60°C for 30 min. After an ultrasound clean treatment, the pTi samples were obtained. The purified sulfur powder (1.5 g) and pTi plates were placed in the furnace for a chemical vapor deposition (CVD) treatment, with the former located in the left heating zone and the latter in the right heating zone. Before heating up, the air in the quartz tube was removed, and argon gas was introduced. The parameters of CVD are as follows: (1) The left heating zone took 37 min to rise to 230°C and maintained the temperature for 1 h; the right heating zone took 37 min to reach 750°C and kept the temperature for 1 h. Argon gas (20 cm^3^/min) was continuously supplied throughout the entire process. The pTi‐TiO_2‐x_ was collected after the temperature cooled to room temperature [33].

Next, WH (20 mg) was re‐suspended in absolute ethyl alcohol (5 mL, 99%) and treated with ultrasound (JY92‐IIN, Ningbo Scientz Biotechnology Co., Ltd., China) for 10 min to thoroughly disperse. Then, it was evenly dropped onto the of pTi‐TiO_2‐x_. After drying at room temperature, the plates were placed in a muffle furnace and heated at a rate of 18°C min^−1^ to 750°C for 3 h, then left to cool naturally. Thus, pTi‐TiO_2‐x_‐WH plates were obtained.

### 4.4 Plasma‐Assisted Physical Vapor Deposition (PVD)

The cleaned pTi plates and the target materials (diameter: 50 mm, thickness: 3 mm) were both placed in the magnetron sputtering system (JGP‐450, Shenyang ZKY Technology Development Co., Ltd., Shenyang, China). After evacuating the chamber to 1×10^−5^ Pa, an appropriate amount of argon gas was added to control the chamber pressure at 1.0 Pa. Then, magnetron sputtering was carried out. After all the target materials reached the glow discharge, they were continuously sputtered for 1 h. Finally, different pTi‐coating samples can be obtained.

### 4.5 Characterization

The filed‐emission scanning electron microscope (FESEM, GeminiSEM 300, ZEISS, Germany) and the transmission electron microscope (TEM,​ JEOL‐2100F, Japan Electronics Co., Ltd, Japan) were used to observe the morphology of the samples. X‐ray photoelectron spectroscopy (XPS, Scientific K‐Alpha, Thermo Scientific, USA) was used to determine the elemental composition of samples. The composition and crystalline phases of the samples were determined by X‐ray diffraction (XRD, D8 Advance, Bruker, Germany). The photoluminescence (PL) spectra were recorded using a spectrofluorometer (Edinburgh, FLS1000, UK) under excitation at 425 nm. AFM results and analysis were achieved by the atomic force microscopy device (Agilent 5500, Bruker Dimension Icon, Germany) and NanoScope Analysis software 3.0. The fluorescence images were obtained through a laser scanning confocal microscope (Olympus, FV3000, Japan). A thermal imager (Testo 875 pro basic, Germany) was used to test the sonothermal properties of the sample. Ultrasound treatments were conducted using an Intelect Mobile Ultrasound (Chattanooga 2776, DJO group, USA).

### 4.6 Theoretical Calculations

Phonon dispersion relations and phonon density of states density were calculated using functional theory calculations. Wavefunctions were expanded using a plane‐wave basis, and the exchange–correlation energy functional was treated within the generalized‐gradient approximation using the Perdew–Burke–Ernzerhof functional. The self‐consistent electronic iterations were converged to within 10^−^^6^ eV, and structural relaxations—including ionic and cell degrees of freedom—were performed until the forces on all atoms were below 0.01 eV/Å. The Gamma ***k***‐mesh methods were used to generate ***k***‐grids, and the ***k***‐resolved was 0.02×2π, the kinetic cutoff energy for the plan‐wave basis was set to 450 eV. Force constants for phonon calculations were obtained via density functional perturbation theory through the VASP code. Phonon dispersion relations and related properties were subsequently calculated form the PHONOPY program [34].

The electronic and thermal transport properties of the heterojunction were investigated through the non‐equilibrium Green's function (NEGF) formalism within the Quantum ESPRESSO package. Electron exchange and correlation were treated with the GGA‐PBE functional, in conjunction with norm‐conserving PseudoDojo pseudopotentials based on a linear combination of atomic orbitals. The valence electrons were extended using a double‐zeta polarized (DZP) numerical atomic orbital basis set for all elements. Electron transmission spectra were computed on a dense 101×101 k‐point grid across the two‐dimensional Brillouin zone. A plane‐wave energy cutoff of 200 Ry was applied. The spin‐resolved ballistic conductance was subsequently determined by integrating the transmission over the Brillouin zone following the Landauer–Büttiker equation [35, 36]:

$$G\left(E\right)=\frac{2e^{2}}{h}T\left(k,E\right)$$

Here T(E) is the Transmission Probability, E Is Energy, and k Is Momentum. The Electronic Thermal Conductance Is Obtained by:

$${K_{\mathrm{el}}}_{,\sigma }=\frac{1}{h}\left[{L_{1}}_{\sigma }\left(E_{F},T\right)eS_{\sigma }+\frac{{L_{2}}_{\sigma }\left(E_{F},T\right)}{T}\right]$$

The *S*, *T, E_F_*, and *e* are seebeck coefficients, temperature of the electron, Fermi level, and electron charge, respectively. The *L (E_F_, T)* is calculated using:

$$-\int d\varepsilon \left\{\partial f\left(\varepsilon ,E_{F},T\right)/\partial \varepsilon \right\}{\left(\varepsilon -E_{F}\right)}^{v}\tau_{\sigma }\left(\varepsilon \right)\left(v=1,2,3\right)$$

The *f (E, E_F_,T)* is the Fermi–Dirac distribution of the Fermi level.

### 4.7 Electron Spin Resonance Detection

The 5,5‐dimethyl‐1‐pyrrolidine‐N‐oxide (DMPO) was selected as a spin‐trapping agent to detect the hydroxyl radical (•OH) generated by the pTi plates with different coatings under US irradiation. In short, pTi, pTi‐TiO_2‐X_, and pTi‐TiO_2‐X_‐WH were respectively placed in the diluted DMPO solution and subjected to US irradiation (1.2 W/cm^2^, 1 MHz, continuous, 5 min/10 min). Then, an equal volume (100 µL) of each solution was separately aspirated into quartz tubes for ESR measurement (JES‐FA200, JEOL Ltd., Japan).

### 4.8 Resistance Testing

Four (I+, I−, V+, V−) copper foil electrodes (KEITHLEY DMM7510, USA) were connected to the coated surfaces of pTi plates with different surface coatings at room temperature of 25°C and ambient humidity of 45%. The 4‐wire mode was selected to test the coating layer. The resistance value of the coating is calculated according to the formula R(resistance) = V(voltage)/I(current) (Ω).

### 4.9 Sonothermal Capability Test

A thermal imager was used to record the temperature changes on the surface of the different coated pTi plates under US irradiation (1.2 W/cm^2^, 1 MHz, continuous, 100% duty cycle). The heating and cooling changes of the surface of the plates with different coatings irradiated by US were also recorded by this thermal imager.

### 4.10 Ultrasonic Electrochemical Measurement

Using the electrochemical analysis equipment (CHI660E, China), under ultrasonic conditions (1.2 W/cm^2^, 1 MHz, continuous), the ultrasonic currents of samples were measured in the Na_2_SO_4_ solution (0.1 M).

### 4.11 Finite‐Element Simulations

Thermo‐acoustic characteristics of coated titanium sheets were numerically investigated in COMSOL 6.3. Ultrasonic waves are launched through the base of the plate and propagate across the titanium substrate before impinging on the rear coating. Distinct coatings yield distinct thermo‐acoustic responses because of their differing thermo‐physical properties, notably thermal conductivity, specific heat and mass density. All simulations were performed in COMSOL Multiphysics 6.3 by solving the coupled governing equations within the built‐in “Heat Transfer in Solids” physics interface. The thermo‐physical properties of each constituent are prescribed as follows. The titanium plate is characterised by a longitudinal sound velocity of 6000 m/s, a mass density of 4500 kg/m^3^, a thermal conductivity of 21 W/m·K and a specific heat capacity of 500 J/kg·K. The Te coating exhibits 2600 m/s, 6200 kg/m^3^, 2 W/m·K and 200 J/kg·K. ZnO is assigned 6300 m/s, 5600 kg/m^3^, 20 W/m·K and 495 J/kg·K. Si domains receive 9000 m/s, 2330 kg/m^3^, 148 W/m·K and 700 J/kg·K. W layers adopt 5000 m/s, 19200 kg/m^3^, 170 W/m·K and 130 J/kg·K. Throughout the simulation the following parameters were held uniform: the diameter and thickness of the titanium plate, the thickness of each coating layer was 10 µm, and a constant ultrasonic output power (1.2 W/cm^2^, 1 MHz).

### 4.12 In Vitro Antibacterial Test

The in vitro antibacterial effect was determined using MRSA (ATCC, Manassas, VA) as the pTi‐TiO_2‐x_‐WH+US. MRSA of 10^7^ CFU/mL was used for evaluation by the spread plate method. In simple terms, the sterile titanium plates (with a diameter of 8.0 mm) obtained by different methods were placed in a 24‐well plate. 1 mL of bacterial solution with a concentration of 10^7^ CFU/ml was added to each well. After incubation at 37°C in an incubator for 48 h, the titanium plates were gently washed three times with sterile PBS to remove floating bacteria, thereby obtaining the biofilm on the surface of the titanium plates. Then, US treatment (1.2 W/cm^2^, 1 MHz, continuous, 15 min or two cycles of 15 min) was performed on pTi and pTi‐TiO_2‐x_‐WH, respectively. During this process, the thermal imager was used to monitor the samples' temperature changes. The MRSA liquids on the sample surfaces were collected and diluted by 20 000‐fold. Then, 20 µL diluted MRSA liquids were spread onto LB agar plates. These spread plates were incubated at 37°C for 24 h. By counting the number of bacterial colonies, the antibacterial rates can be calculated with the following equation:

$$\mathrm{Antibacterial}\;\mathrm{Ration}\left(\%\right)=\left(\mathrm{NCtrl}-\mathrm{NSample}\right)/\mathrm{NCtrl}\times 100\%$$

The live/death (green/red) status of MRSA on the surface of the sample was observed using a laser confocal microscope. The bacteria were stained using the Viability/Cytotoxicity Assay Kit for bacterial cells (L6060S, UElandy, China), and then observed under the laser confocal microscope.

### 4.13 Isolation and Culture of hBMSCs

hBMSCs were extracted from the bone marrow blood of healthy volunteer donors, which were obtained from the Department of Orthopedics, Wuhan Union Hospital, China, according to the protocol of the Ethics Committee of Tongji Medical College, Huazhong University of Science and Technology (No. S347). The collected bone marrow blood was mixed with sterile phosphate buffer saline (PBS) at a ratio of 1:2. Human lymphocyte isolation solution was then added in a 1:1 volume, followed by centrifugation to obtain hBMSCs. The obtained hBMSCs were seeded into Dulbecco's modified eagle medium (DMEM)/F12 medium (Gibco) containing 10% fetal bovine serum (FBS). It was cultivated in an incubator at 37°C and 5% CO_2_, and the culture medium was replaced every two days.

### 4.14 CCK‐8 Assay

First, sterile titanium plates with different surface coatings were placed in different 48‐well plates. hBMSCs were seeded in a number of 1 × 10^4^ per well in a 48‐well plate and co‐cultured with Ti plates with different coatings, and CCK‐8 assay was performed on Day 1, 3, and 7. The medium in each well was discarded and washed with PBS. 300 µL medium containing 10% CCK‐8 was added to each well and placed in a 37°C and 5% CO_2_ incubator for 2 h. Then 100 µL supernatant was aspirated and transferred to a 96‐well plate to avoid bubble formation. The absorbance at 450 nm was recorded with a microplate reader.

To study the effect of US on the viability of hBMSCs, hBMSCs were first co‐cultured with pTi‐TiO_2‐x_‐WH plates, and the parameters of US were: 0.2 W/cm^2^, 1.0 MHz, 50% duty cycle for 10 min. In all cell experiments in this study, the thickness of the coupler was 3 cm. CCK‐8 assay was performed on Days 1, 3, and 7.

### 4.15 Alizarin Red S Staining

Sterile pTi plates with different surface modifications were placed in 48‐well plates, and 1 × 10^4^ hBMSCs were inoculated into each well for co‐culture for 21 days. During the induction of osteogenic differentiation, the pTi‐TiO_2‐x_‐WH group was treated with US irradiation once every 3 days for 15 min each time. On day 21, the supernatant was discarded, washed 3 times with PBS, and fixed in 4% paraformaldehyde for 30 min. An appropriate amount of 0.2% Alizarin Red S staining solution (ALIR‐10001, OriCell) was added and stained at room temperature for 30 min in the dark. After washing again with PBS, the formation of calcium nodules on the surface was observed. Then, 1 mL Cetylpyridinium Chloride Monohydrate (IC0280, Solarbio, Beijing, China) containing 10% was added to each well. After 15 min, 100 µL liquid was absorbed into a 96‐well plate, and its OD_562 nm_ was determined.

### 4.16 Quantitative Real‐Time Polymerase Chain Reaction (qRT‐PCR)

The following groups were tested: pTi, pTi‐TiO_2‐x_‐WH, and pTi‐TiO_2‐x_‐WH+US. hBMSCs were seeded in different groups of 6‐well plates and incubated for 14 days under the corresponding conditions. The pTi‐TiO_2‐x_‐WH+US group was treated with US (0.2 W/cm^2^, 1.0 MHz, 50% duty cycle for 10 min) every three days. After this treatment, the RNA was extracted using the FastPureCell/Tissue Total RNA Isolation Kit V2 (Vazyme Biotech Co., Ltd) according to the manufacturer's instructions. The concentration of RNA was measured using an ultramicrospectrophotometer. HiScript III RT SuperMix (Vazyme, R323‐01) was added to the RNA, and the mixture was reverse transcribed into cDNA (Applied Biosystems). The prepared primers, cDNA, DEPC water (BL510A, Biosharp), and SYBR fluorescent dye (Q712‐02, Vazyme) were mixed and transferred to an enzyme‐free 96‐well plate. The CFX ConnectTM real‐time system (BioRad Laboratories, USA) was used to detect each target gene. The obtained Cq values were normalized using GAPDH, and the gene expression of each group was calculated using the 2^−ΔΔ^CT method.

### 4.17 RNA Sequencing

hBMSCs were divided into pTi group, pTi‐TiO_2‐X_‐WH group, and pTi‐TiO_2‐X_‐WH+US group. The cells were incubated in corresponding 6‐well plates for 14 days. Total RNA from each group of hBMSCs was extracted using TRIzol reagent. RNA samples were quantified after integrity and quality control checks, and then an RNA sequencing library was built. Data were processed using Illumina Novaseq 6000 PE150 (Illumina, USA) with the assistance of OBiO Technology Co., Ltd (Shanghai, China). EdgeR software (v3.28.1) was used for analysis. Differentially expressed genes were subjected to GO analysis and KEGG enrichment analysis with a *p*‐value below 0.05.

### 4.18 Immunofluorescence Staining

hBMSCs were seeded in 48‐well plates, and the groups were set as: pTi group, pTi‐TiO_2‐X_‐WH group, and pTi‐TiO_2‐X_‐WH +US group. During the two‐week period of cell culture, US treatment (0.2 W/cm^2^, 1.0 MHz, 50% duty cycle, 10 min) was applied once every three days. The cells were first fixed with 4% paraformaldehyde for 30 min, permeabilized with 0.3% Triton X‐100 at room temperature for 15 min, and washed three times with PBS. After blocking with normal goat serum for 30 min, the corresponding primary antibodies were added, and the samples were incubated overnight at 4°C. The next day, the cells were washed three times with PBS containing 0.1% Tween‐20, incubated with the species‐appropriate Alexa‐Fluor‐594‐conjugated secondary antibody at room temperature for 60 min, and washed again. Nuclei and F‐actin were then counter‐stained with DAPI (Invitrogen, USA) and YF 488–phalloidin (Uelandy, China), respectively, and fluorescence images were acquired on a confocal microscope.

### 4.19 In Vitro Thrombolysis Assay

Male SD rats (180–220 g) were anesthetized, and blood was collected via cardiac puncture. The blood was immediately injected at a constant rate into siliconized tubes (1 mm inner diameter), which were then sealed at both ends and incubated in a 37°C water bath for 3 h to allow clot formation. The thrombus was then gently extruded, rinsed three times with ice‐cold PBS to remove residual blood, cut into uniform 0.5‐cm segments, and weighed. Individual thrombus segments were placed into separate wells of a 24‐well plate and randomly divided into six groups: PBS group, PBS+US group, urokinase (UK) group, UK+US group, NiTi‐TiO_2‐x_+US group, and NiTi‐TiO_2‐x_+UK+US group. US treatment (0.7 W/cm^2^, 1 MHz, continuous) was performed for 10 min, and all groups were then incubated at 37°C. At the endpoint, residual thrombi were weighed, and supernatant absorbance was read at 450 nm on a microplate reader.

### 4.20 Animal Experiment

The male Sprague‐Dawley (SD) rats (180–220 g) were selected from the Laboratory Animal Center of Huazhong University of Science and Technology as the in vivo experimental model. The feeding environment was in line with SPF grade. Rats and Beagles experiments were approved by the Animal Research Committee of Huazhong University of Science and Technology, Wuhan, China (No. 4744) and Wuhan Deyuan Biotechnology Co., Ltd. (DYSW251107), respectively. Rats were randomly assigned to six groups: three infected (pTi, pTi+US, and pTi‐TiO_2‐x_‐WH+US) and three non‐infected groups (MRSA‐pTi+US, MRSA‐pTi‐TiO_2‐x_‐WH, MRSA‐pTi‐TiO_2‐x_‐WH+US two‐cycle). The infected screws were immersed in MRSA solution for 2 days (37°C) and formed biofilm before insertion. After intraperitoneal anesthesia with 3% pentobarbital (30 mg/kg), the right lower limb was depilated and disinfected. The scalpel was used to make a longitudinal incision of the skin on the lateral side below the right knee joint, exposing the subcutaneous tissue, separating the muscle and soft tissue, and exposing the right tibial tubercle. Locate the nail point, use 1.2 mm drill to open the hole, screw in the corresponding screws according to the designed group, and suture the incision. On the third postoperative day, the US group received US treatment (1.2 W/cm^2^, 1 MHz, continuous, two cycles of 15 min). The rats were sacrificed on the 28th day after treatment, and the tibia with screws, heart, liver, spleen, lung, and kidney were harvested.

Beagle dogs with thrombosis were randomly divided into three groups: Control group, UK group, and NiTi‐TiO_2‐x_+UK+US group. Following intramuscular injection of Zoletil (Virbac, France, 20 mg/kg), the right inguinal area was shaved and disinfected with povidone‐iodine. A longitudinal incision was made in the right inguinal region along the course of the iliac artery to expose the right femoral vein. After dissecting the vein free, a 2.5‐cm segment was isolated and ligated at both proximal and distal ends with silk sutures to interrupt blood flow, followed by layered closure of the skin. At 48 h postoperatively, the ultrasonography of the right femoral vein was performed to confirm thrombus formation. After confirmation, the distal ligation suture was removed. In the UK group, 100,000 units of UK was injected into the thrombus; in the NiTi‐TiO_2‐x_+UK+US group, a NiTi‐TiO_2‐x_ guidewire was inserted into the thrombus and followed by the injection of UK. After that, the ultrasound treatment (1.2 W/cm^2^, 1 MHz, continuous, 10 min) was administered. Thirty minutes later, thrombus status was evaluated by ultrasonography. Finally, the femoral vein segment at the thrombosis sites was harvested, fixed in 4% PFA, and stained with H&E.

### 4.21 In Vivo Antibacterial Test

To further test the in vivo antibacterial ability of pTi‐TiO_2‐X_‐WH+US, the experiments were divided into pTi group and pTi‐TiO_2‐x_‐WH+US group. The screws in both groups were immersed in MRSA bacteria solution for 2 days (37°C) to form biofilms, and then the screws were placed into the right tibia of the rats as described above. After the incision was sutured, both groups of screws were treated with US (1.2 W/cm^2^, 1 MHz, continuous, 15 min, two cycles). After US treatment, the skin at the incision was opened, and the screw was removed. The MRSA on the screw surface was spread plate.

### 4.22 Histological Analysis

The heart, liver, spleen, lung, and kidney were stained with H&E. After dehydration, the bone tissue of the tibia was embedded in Methyl methacrylate (MMA) (EXACT E520, Germany), hard tissue sections (Leica SP1600, Germany), and grinding pieces (minimet 1000, Buehler, USA). Then the sections were stained with Van Gieson (VG).

### 4.23 Micro‐CT

The tibia at the screw insertion site was scanned using Micro‐CT (Bruker, Skyscan1176), and 3D digital images were constructed and BV/TV values were obtained using SkyScan CT and CT‐Vox software.

### 4.24 Statistical Analysis

The experimental data were analyzed using Origin 2021 and GraphPad Prism 8 software. The results were presented as the mean ± standard deviation. All experiments were conducted more than three times. One‐way ANOVA followed by Tukey's multiple‐comparisons test was used for multiple groups. It is considered that *p* > 0.05 has no significance (ns), while ^*^ *p* < 0.05, ^**^ *p* < 0.01, ^***^ *p* < 0.001, and ^****^ *p* < 0.0001 are considered to have statistical significance. The researchers were unaware of the group allocation during the data collection and data analysis phases. No data or samples were excluded from the study.

## Conflicts of Interest

The authors declare no conflicts of interest.

## Supporting information




**Supporting File**: advs77206‐sup‐0001‐SuppMat.docx.

## Data Availability

The data that support the findings of this study are available on request from the corresponding author. The data are not publicly available due to privacy or ethical restrictions.
