# Supplementary material for: Electronic Chip‐Mimetic Medical Implants for Controllable Sonothermal Treatments
Source: Adv Sci (Weinh). 2026 Aug 15;13(61):e77206. doi: 10.1002/advs.77206 (PMC13477257; doi:10.1002/advs.77206)
Supplement: Supplementary file 1 [file ADVS-13-e77206-s001.docx]

Supporting Information

**Electronic chip-mimetic medical implants for controllable sonothermal treatments**

Zhengdong Zhang^1,2,#^, Pan Liu^1,3,#^, Jie Lei^1,4,#^, Shuchang Peng^1,#^, Qiu Chen^5^, Wei Guan^6^, Yizhou Zhu^6^, Gang Liu^2,^*, Xiaobo Feng^1,^*, Cao Yang^1,^*, Lei Tan^1,^*

Corresponding author

*Correspondence to: Lei Tan [(tanlei_hubu@163.com)](mailto:(tanlei_union@hust.edu.cn)); Cao Yang [(caoyangunion@hust.edu.cn)](mailto:(caoyangunion@hust.edu.cn)); Xiaobo Feng (fengxiaobo@hust.edu.cn); Gang Liu (liugang1969cyfy@163.com).

The file includes:

1. Figure S1-S14

2. Table S1

**Figure S1.** The SEM and element mapping images of pTi, pTi-W, pTi-Fe, pTi-Mo, pTi-Zn, pTi-TiO_2_, pTi-Si, pTi-ZnO, and pTi-Te (pTi-Te-2 in Figure 3b).

**Figure S2.** The bandgaps of pTi-Si, pTi-TiO_2_, pTi-ZnO, and pTi-Te.

**Figure S3.** DFT-simulated heterojunction interface structures of Ti-W, Ti-Si, Ti-ZnO, and Ti-Te.

**Figure S4**. DFT-simulated unit cell structures of Ti, W, ZnO, Si and Te.

**Figure S5.** DFT-simulated group velocities of Ti, W, Si, ZnO and Te.

**Figure S6.** Images of pTi-TiO_2-x_ and pTi-TiO_2-x_-WH plates and the cyclic heating and cooling curve of pTi-TiO_2-x_-WH.

**Figure S7.** (a) Bandgap of pTi-TiO_2-x_ and (b) Nanoindentation results of pTi-TiO_2-x_ and pTi-TiO_2-x_-WH.

**Figure S8.** XPS of pTi-TO_2-x_ and pTi-TO_2-x_-WH.

**Figure S9.** The piezoelectric hysteresis loops of pTi-WH.

**Figure S10.** The amplitude and phase images of pTi-TO_2-x_-WH and pTi-WH.

**Figure S11**. The MTT assay of MSCs co-cultured with Control (Ti), pTi, pTi-TO_2-x_, pTi-TO_2-x_-WH and pTi-TO_2-x_-WH+US for 1, 3, and 7 days. The ultrasonic time is 15 min. n = 3 independent experiments per group, data are expressed as mean ± SD.

**Figure S12**. (a) Heatmap of differentially expressed genes of pTi vs pTi-TO_2-x_-WH+US. (b) Gene set enrichment analysis of pTi vs pTi-TO_2-x_-WH+US.

Figure S13. Immunohistochemical staining of Coll I and Piezo1.

**Figure S14.** The H&E staining of the main organs of rats.

**Table S1.** The primer sequences of the osteogenesis-related genes.

| Genes | Primer sequences |  |
| --- | --- | --- |
| GAPDH | 5'-GGCAAGTTCAACGGCACAGT-3' (forward) | 5'-GCCAGTAGACTCCACGACAT-3' (reverse) |
| ALP | 5'-GTCCCACAAGAGCCCACAAT-3' (forward) | 5'-CAACGGCAGAGCCAGGAAT-3'(reverse) |
| COL1 | 5'-CTGCCCAGAAGAATATGTATCACC-3' (forward) | 5'-GAAGCAAAGTTTCCTCCAAGACC-3' (reverse) |
| OCN | 5'-CAGTAAGGTGGTGAATAGACTCCG-3' (forward) | 5'-GGTGCCATAGATGCGCTTG-3' (reverse) |
| RUNX2 | 5'-CCGAGACCAACCGAGTCATT-3' (forward) | 5'-CACTGCACTGAAGAGGCTGT-3' (reverse) |
